# Characterization and Genomic Analysis of PALS2, a Novel *Staphylococcus* Jumbo Bacteriophage

**DOI:** 10.3389/fmicb.2021.622755

**Published:** 2021-03-08

**Authors:** Yoona Lee, Bokyung Son, Yoyeon Cha, Sangryeol Ryu

**Affiliations:** ^1^Department of Food and Animal Biotechnology, Seoul National University, Seoul, South Korea; ^2^Department of Agricultural Biotechnology, Seoul National University, Seoul, South Korea; ^3^Research Institute of Agriculture and Life Sciences, Seoul National University, Seoul, South Korea; ^4^Center for Food and Bioconvergence, Seoul National University, Seoul, South Korea

**Keywords:** *Staphylococcus aureus*, jumbo phage, antimicrobial agent, antibiotic resistance, genome

## Abstract

*Staphylococcus aureus* is an important human pathogen that can be frequently encountered in clinical and food-processing surroundings. Among the various countermeasures, bacteriophages have been considered to be promising alternatives to antibiotics. In this study, the bacteriophage PALS2 was isolated from bird feces, and the genomic and biological characteristics of this phage were investigated. PALS2 was determined to belong to the *Myoviridae* family and exhibited extended host inhibition that persisted for up to 24 h with repeated bursts of 12 plaque-forming units/cell. The complete genome of PALS2 measured 268,746 base pairs (bp), indicating that PALS2 could be classified as a jumbo phage. The PALS2 genome contained 279 ORFs and 1 tRNA covering asparagine, and the majority of predicted PALS2 genes encoded hypothetical proteins. Additional genes involved in DNA replication and repair, nucleotide metabolism, and genes encoding multisubunit RNA polymerase were identified in the PALS2 genome, which is a common feature of typical jumbo phages. Comparative genomic analysis indicated that PALS2 is a phiKZ-related virus and is more similar to typical jumbo phages than to staphylococcal phages. Additionally, the effective antimicrobial activities of phage PALS2 suggest its possible use as a biocontrol agent in various clinical and food processing environments.

## Introduction

*Staphylococcus aureus* is a Gram-positive bacterium that causes skin infections, respiratory tract infections, and food poisoning in animals and the human body (Lowy, 1998; De Lencastre et al., 2007). Staphylococcal infection can be life-threatening if not treated immediately. Moreover, treatment of the infection has become more difficult because of the emergence of antibiotic-resistant bacteria. In particular, methicillin-resistant *S. aureus* (MRSA) is more challenging than other resistant bacteria, as it is resistant to the entire classes of beta-lactam antibiotics and even to multiple classes of non–beta-lactam antibiotics (Chambers and DeLeo, 2009; Turner et al., 2019). Additionally, MRSA ranked high on the World Health Organization’s priority list for the development of new antibiotics for antibiotic-resistant bacteria (Khan et al., 2019). These findings highlight the need to devise a new strategy to combat *S. aureus*, and there has been increasing interest in the development of alternative antimicrobial agents employing bacteriophages (phages) (Foster, 2004).

Phages are a type of virus that specifically infects bacteria, and they have several potential advantages over traditional antibiotics, such as specificity to target bacteria, capacity to self-replicate, and safety. A phage follows one of two life cycles, lytic and lysogenic cycles, and the phages that obligatorily follow the lytic cycle are better candidates for therapeutic use. Lytic phages that infect their hosts rapidly replicate and produce many progenies and ultimately lyse the bacteria, which are preferred in comparison to the phages that integrate their genome into the bacterial genome (Ghannad and Mohammadi, 2012; Altamirano and Barr, 2019; Cui et al., 2019; Reuter and Kruger, 2020).

Genomic sequence analysis revealed that phages are diverse in their genome size. Iyer et al. (2021) classified phages into three groups: small phages with genome sizes less than 100 kb, medium-sized phages with genome sizes less than 180 kb, and phages with genomes larger than 180 kb. The majority of phages reported to date have genome sizes less than 200 kbp referred to as small-genome phages, and phages with genome sizes above 200 kbp but below 500 kbp have mostly been classified as jumbo phages (Yuan and Gao, 2017). The large genome size of jumbo phages enables many proteins that rarely or do not exist in small-genome phages to be encoded, and these proteins are more complex than those existing in small-genome phages. For instance, several small-genome phages such as phage T7 have its own RNAP, but it is a single subunit RNAP, whereas most jumbo phages encode a multisubunit RNAP (Sokolova et al., 2020). This feature makes the jumbo phages generally less dependent on the host metabolism and eventually leads them to have a wider host range compared to the phages with smaller genomes (Mesyanzhinov et al., 2002). Accordingly, these attributes make jumbo phages ideal for phage therapeutic application (Guan and Bondy-Denomy, 2020). However, jumbo phages have been rarely isolated by classical phage isolation method because of the large size of virions. Jumbo phages have mostly been isolated from Gram-negative bacteria, and only 11 jumbo phages have been reported from Gram-positive bacteria to date, most of which are *Bacillus*-infecting phages and one *Staphylococcus* infecting phage, S6 (Uchiyama et al., 2014; Lood et al., 2020). *Staphylococcus* Jumbo phage S6 belongs to *Myoviridae* with genome size of approximately 270 kbp, but its genome has not been sequenced. Besides, the isolated jumbo phages contain a variety of genes with unknown functions, indicating that the jumbo phage genomes represent extremely high genetic diversity and that their functional biological characteristics have not been fully elucidated. Therefore, further research is required to obtain more fundamental understanding of jumbo phages.

In this study, we isolated a novel staphylococcal jumbo phage, PALS2 (accession no. MN091626), which has a genome size of 268,748 base pairs (bp). We investigated the morphology, host infectivity, and bioinformatics characteristics of this phage. Interestingly, PALS2 exhibited multiple small bursts to efficiently generate progeny phages and presented several common genomic features of the jumbo phages. Considering the strong bacterial inhibitory ability and broad host range of PALS2, this phage could be utilized in the development of a novel potential biocontrol agent (Hyman, 2019).

## Materials and Methods

### Bacterial Strains and Growth Conditions

The bacterial strains used in this study are listed in Table 1. *S. aureus* human isolate strain 93 was used for the isolation and propagation of the phage PALS2. All staphylococcal and enterococcal strains were grown in tryptic soy broth (TSB), and other bacteria were grown in Luria–Bertani (LB) broth at 37°C.

**TABLE 1:** Antimicrobial spectrum of phage PALS2 against different bacterial species.

Bacterial host^a^	PALS2^b^
**Staphylococcal strain**	
*Staphylococcus aureus* RN 4220	T
*S. aureus* Newman	C
*S. aureus* ATCC 13301	C
*S. aureus* ATCC 23235	T
*S. aureus* ATCC 33586	T
*S. aureus* ATCC 33593	C
*S. aureus* ATCC 6538	C
*S. aureus* ATCC 29213	C
*S. aureus* ATCC 12600	T
*S. aureus* ATCC 25923	I
*S. aureus* ATCC 27664	T
MRSA CCARM 3793	C
MRSA CCARM 3089	T
MRSA CCARM 3090	T
*Staphylococcus haemolyticus* ATCC 29970	C
*Staphylococcus epidermidis* ATCC 35983	I
MRSE CCARM 3787	I
MRSE CCARM 3789	I
*Staphylococcus hominis* ATCC 37844	C
*Staphylococcus warneri* ATCC 10209	T
*Staphylococcus xylosus* ATCC 29971	I
*Staphylococcus saprophyticus* ATCC 15305	T
*Staphylococcus capitis* ATCC 35661	C
*Staphylococcus cohnii* ATCC 29974	C
**Other Gram-positive bacteria**	
*Enterococcus faecalis* ATCC 29212	–
*Bacillus cereus* ATCC 14579	–
*Bacillus subtilis* ATCC 23857	–
*Listeria monocytogenes* ATCC 19114	–
**Gram-negative bacteria**	
*Salmonella enterica* serovar Typhimurium SL 1344	–
*Escherichia coli* MG1655 ATCC 47076	–
*Cronobacter sakazakii* ATCC 29544	–
*Pseudomonas aeruginosa* ATCC 27853	–

*^*a*^ATCC, American Type Culture Collection. ^*b*^C, clear plaque; T, turbid plaque; I, turbid lysis zone without single plaques (Supplementary Figure 1); –, no lytic effect.*

### Bacteriophage Isolation and Propagation

Bacteriophage PALS2 was isolated from bird feces. Bird fecal samples were collected in Seoul, South Korea. A sample mixed with 50 mL of sodium chloride (NaCl)–magnesium sulfate (SM) buffer (50 mM Tris–HCl, pH 7.5, 100 mM NaCl, and 8 mM MgSO_4_⋅7H_2_O) was poured into a stomacher filter bag and homogenized. From the other side of the filter in the bag, the sample was transferred to a 50 mL tube and centrifuged at 10,000 × *g* for 5 min at 4°C. After centrifugation, the supernatant was filtered using a 0.22 μm polyethersulfone (PES) membrane filter to remove bacterial cells. Then, 5 mL of the filtrate was added to an equal volume of 2 × TSB broth and subcultured with the host strain at 37°C with shaking at 220 revolutions/min (rpm) for 12 h. After incubation, the culture was centrifuged, and the supernatant was filtered as described above. The presence of phage in the filtrate was confirmed by spotting 10 μL of 10-fold serially diluted filtrates onto soft agar (TSB containing 0.4% agar) containing 100 μL of host *S. aureus* culture. The plates were incubated overnight at 37°C, and the formation of plaques was monitored. Based on the typical plaque morphology of the jumbo phage, small plaques were picked with a sterile tip and eluted in 100 μL SM buffer (Serwer et al., 2007; Lewis et al., 2020). The plaque isolation and elution steps were repeated more than three times to purify a single phage; phages were incubated at 30°C.

For propagation of the phage, TSB was first inoculated with the host *S. aureus* strain and incubated at 37°C with shaking at 220 rpm until it reached an OD_600_ of 0.25. Subsequently, CaCl_2_ and MgCl_2_ (at final concentrations of 5 mM each) and phages at a multiplicity of infection (MOI) of one were added followed by a 4 h incubation at 30°C. The propagated phages were centrifuged at 15,000 × *g* for 5 min, and the supernatant was filtered using a 0.22 μm PES filter to remove bacterial debris. To prepare the phage at a high titer, filtered phages of 40 mL were mixed with 5 g of polyethylene glycol 6000 and 5 mL of 5 M NaCl using a two-dimensional shaker at 4°C overnight. Phages were then precipitated by centrifugation (15,000 × *g*; 20 min; 4°C), and the pellet suspended in SM buffer was subjected to a four-layer CsCl gradient (1.3, 1.45, 1.5, and 1.7 g/mL). After ultracentrifugation at 25,000 × *g* for 2 h at 4°C, the separated phage was dialyzed against 1 L of dialysis buffer (50 mM Tris–HCl, pH 8.0, 10 mM NaCl, and 10 mM MgCl_2_) using a dialysis membrane tube for 2 h with a buffer change.

### Morphological Analysis by TEM

*Staphylococcus* phage PALS2 was analyzed using transmission electron microscopy (TEM). A phage suspension [1 × 10^10^ plaque-forming units (PFUs)/mL] was placed on a carbon-coated copper grid and negatively stained with 2% uranyl acetate (pH 4.0). The sample was examined with an energy-filtering TEM at an operating voltage of 120 kV (Kwiatek et al., 2012). PALS2 was identified and classified according to the guidelines of the International Committee on Taxonomy of Viruses (Lefkowitz et al., 2018).

### Bacterial Challenge Assay

Tryptic soy broth (50 mL) was inoculated with the host strain *S. aureus* human isolate 93 and incubated at 37°C until the early exponential growth phase was reached. The culture was subsequently infected with the phage at MOIs of 1 and 0.1. Bacterial growth was monitored by measuring the optical density at 600 nm (OD_600_) each hour after phage infection, with no measurements being taken between the 7th and 16th hours (Park et al., 2012). An uninfected culture was used as a negative control.

### Host Range Analysis

The bacterial strains listed in Table 1 were incubated overnight at 37°C. One hundred microliters of each bacterial culture was mixed with 5 mL of soft agar (TSB containing 0.4% agar) and overlaid on tryptic soy agar plates. Subsequently, 10 μL of serially diluted phage PALS2 lysates (tenfold, 10^10^ to 10^3^ PFUs/mL) was spotted onto the prepared plates and incubated at 30°C overnight. After incubation, the infectivity was determined based on the appearance of the spots: “C,” clear single plaques; “T,” turbid single plaques; “I,” inhibited growth without single plaques; “—,” no lysis nor growth inhibition.

### Receptor Analysis

To verify the receptor, *S. aureus* RN4220, a strain free of prophages (Kreiswirth et al., 1983), type I restriction mechanisms, and capsules (Wann et al., 1999), was utilized (Table 1). It is known that most genetic manipulation of *S. aureus* is confined to strain RN4220 because RN4220 is defective of type I restriction–modification (RM) system, whereas different lineages of *S. aureus* contain a unique pair of type I RM systems (Cooper et al., 2017). We obtained an RN4220Δ*tagO*:erm mutant, which lacks the peptidoglycan-anchored wall teichoic acid (WTA) (Swoboda et al., 2010), and its complemented strain carrying the pS*tagO* plasmid. This plasmid was constructed by subcloning the *tagO* gene into an *Escherichia coli–S. aureus* shuttle expression vector, pND50 (Brückner, 1992). Afterward, 10 μL of 10-fold diluted phage PALS2 lysate (10^10^ to 10^3^ PFUs/mL) was spotted from top–left to bottom–right on soft agar (TSB containing 0.4% agar), beginning with the highest titer, with the wild-type RN4220, the RN4220Δ*tagO*:erm mutant, and the *tagO*-complemented strain.

### One-Step Growth Curve

A one-step growth curve analysis was performed as described previously (Lu et al., 2003). Briefly, phage was mixed with the *S. aureus* host strain in the early exponential growth phase (5.6 × 10^7^ colony-forming units/mL) at an MOI of 0.1. After incubation at 37°C for 5 min to enable adsorption of the phage, it was centrifuged at 10,000 × *g* for 5 min. The pellet containing infected cells was resuspended in 50 mL of fresh TSB and incubated at 37°C with shaking at 220 rpm. Samples were collected every 10 min for 6 h. Subsequently, each sample was centrifuged at 16,000 × *g* for 1 min, and the sample titer was assessed by spotting tenfold serially diluted supernatants on a double-layer agar plate. Latent period and burst size were analyzed according to the resulting titer. The latent period of phage was calculated by measuring the interval of time between phage infection and the initial burst of phage titers. The burst size of phage was calculated by dividing the phage titers at postburst plateau phase by the initial phage titers.

### Phage DNA Extraction

Bacteriophage genomic DNA was purified as previously described (Wilcox et al., 1996). Prior to purification, the phage lysate was treated with DNase and RNase for 30 min at room temperature to remove bacterial nucleic acid contaminants. To degrade the phage capsid, phage lysates were then treated with lysis buffer containing 20 mM EDTA, 50 μg/mL proteinase K, and 0.5% sodium dodecyl sulfate for 1 h at 56°C. Phenol–chloroform–isoamyl alcohol (25:24:1) was added to the mixture at a 1:1 ratio and was gently inverted. The resulting sample was centrifuged at 5,000 rpm for 5 min. The top aqueous layer was collected and treated with a 1:1 mixture of equilibrated phenol and chloroform at a 1:1 ratio. The solution was mixed, centrifuged, and isolated as described above. These steps were repeated after treating the isolated top solution with chloroform. Finally, ethanol precipitation was performed. The DNA pellet was washed with 100% ethanol and resuspended in TE buffer.

### Full Genome Sequencing and Bioinformatics Analysis

Purified PALS2 phage genomic DNA was sequenced using the Illumina MiSeq system and assembled with the *de novo* assembly algorithm of CLC Genomics Workbench 10.0.1 at Sanigen Inc., South Korea. Open reading frames (ORFs) were predicted using the Glimmer v3.02 (Delcher et al., 2007) and GeneMarkS (Besemer et al., 2001) software packages. The ORFs were annotated using the Rapid Annotation using Subsystem Technology pipeline, as described previously (McNair et al., 2018). This annotation was further complemented using BlastP (Altschul et al., 1997) and Cognizer (Bose et al., 2015). The complete genome sequence of *Staphylococcus* phage PALS2 was deposited in GenBank under the accession number MN091626.

To map phage PALS2 onto the phage population network, vContact2 (Jang et al., 2019) was utilized, which extracts predicted proteins from each viral genome to build viral protein clusters (PCs), which are subsequently utilized to calculate genome similarities between pairs of viruses. This approach was utilized because viruses lack a universal gene marker. In fact, the coding genes for terminase large subunits, which could have played potential roles as phylogenetic markers because of their relative conservedness to other phage proteins, were not predicted to be present in the genome of phage PALS2. Briefly, the phage PALS2 genome was processed using VirSorter (v.1.03) and vContact2-Gene2Genome (v.1.1.0) on the CyVerse cyberinfrastructure (Merchant et al., 2016) prior to using vContact2. Next, using NCBI Bacterial and Archeal Viral RefSeq (v.88) as the reference database, protein sequences were subjected to all-to-all BlastP searches with an *E* value threshold of 10^–4^ and were defined as homologous PCs in the same manner as previously described (Bolduc et al., 2017). Based on the number of shared PCs between the genomes, vContact2 calculated the degree of similarity as the negative logarithmic score by multiplying the hypergeometric similarity *p* value by the total number of pairwise comparisons. Subsequently, pairs of closely related genomes with a similarity score of ≥1 were grouped into viral clusters (VCs) using ClusterONE (Nepusz et al., 2012) equipped in the vContact2 tool. The resulting network was visualized with Cytoscape (v.3.7.2) using an edge-weighted spring embedded model, which places the genomes or fragments sharing more PCs closer to one another.

## Results and Discussion

### Morphological Characteristics of Phage PALS2

The new *S. aureus*–infecting phage PALS2 was isolated from bird feces. This phage formed relatively small clear plaques (<1 mm on 0.4% softer agar) on the lawn of *S. aureus* human isolate 93 as a bacterial host strain, indicating that PALS2 possesses a characteristic common to large phages, such as jumbo phages (Saad et al., 2019). A previous study observed that virion diffusion of large phages is retarded on a top agar layer, resulting in the formation of small plaques (Gallet et al., 2011).

**FIGURE 1:**
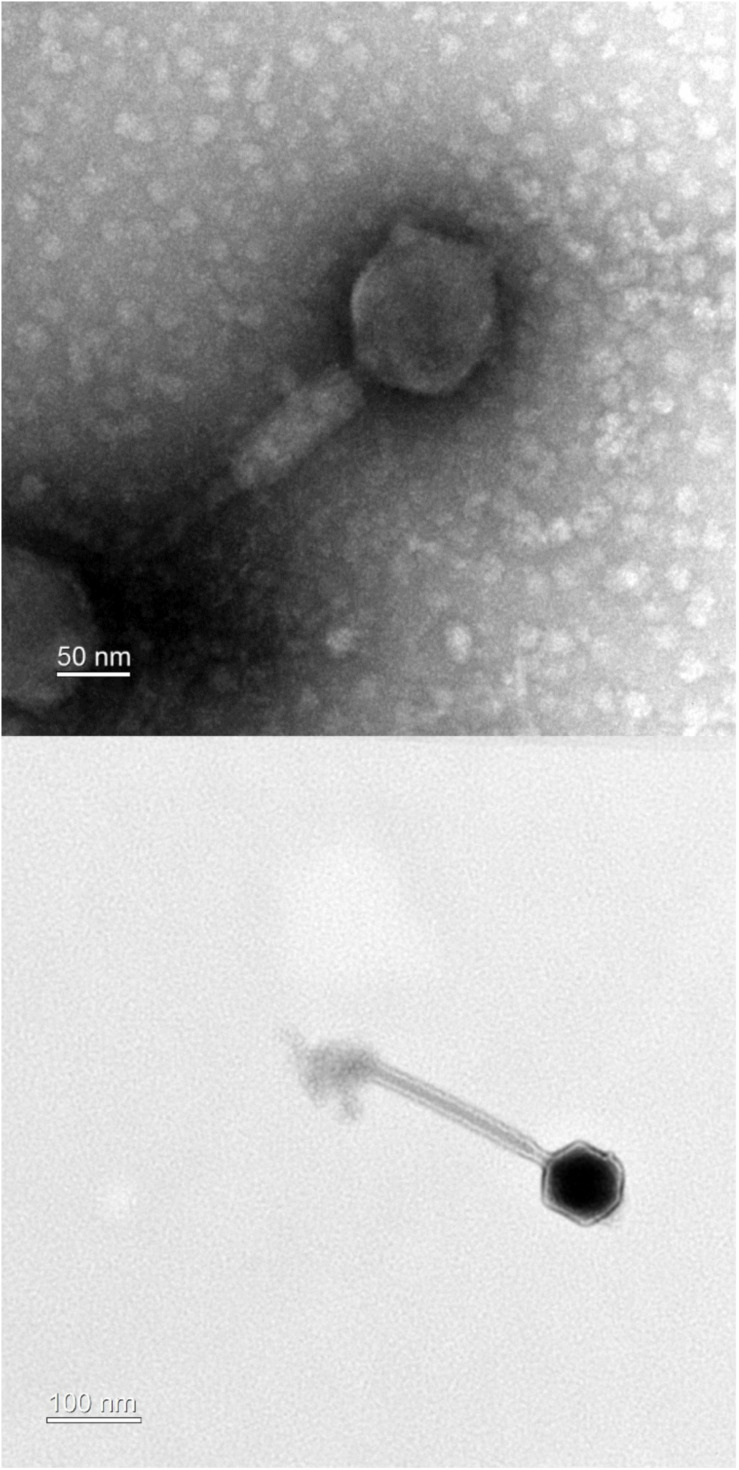
Transmission electron microscopy of phage PALS2. The phage belongs to the family *Myoviridae.*

Transmission electron microscopy analysis of purified PALS2 demonstrated that the phage possesses an icosahedral head with a non-flexible and contractile tail, indicating that this phage belongs to the *Myoviridae* family. The diameters of the head measured approximately 94 nm in width and 101 nm in height. The contractile tail was 201 ± 29 nm in length (*n* = 4 phages) (Figure 1). The basal tuft extending from the baseplate also supported that this phage has the morphotype of a myovirid (O’Flaherty et al., 2005). Of more than a hundred jumbo phages reported to date, approximately 90% have been classified as *Myoviridae* (Yuan and Gao, 2017; Imam et al., 2019).

### Bacterial Challenge Assay of Phage PALS2

**FIGURE 2:**
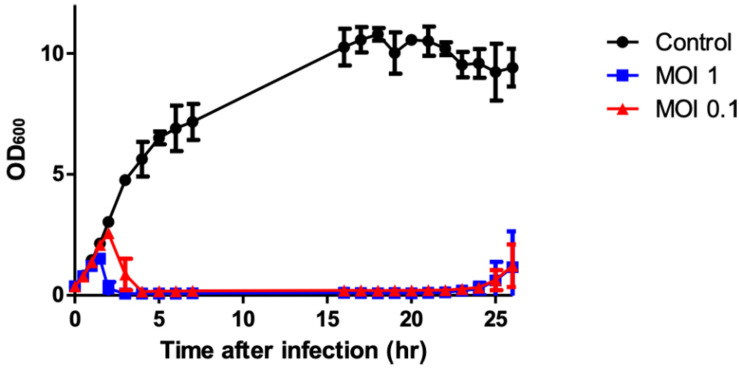
Bacterial growth inhibition assay against *S. aureus*. Cells were prepared in three groups: the control group without phage (∙) and the experimental group with phage (■, ▲; MOIs of 1 and 0.1, respectively). The data shown are the mean values from three independent measurements, and the error bars represent the standard deviations.

A bacterial challenge assay was performed in liquid culture to determine the bacterial growth inhibition by phage PALS2. The complete inhibition of host cells by PALS2 was observed 1.5 or 2 h after infection with MOI 1 or 0.1, respectively, and persisted for up to 24 h after infection with both MOIs (Figure 2). These results demonstrate that PALS2 has a strong bacterial inhibitory ability against its target host bacteria. *S. aureus* phages SA97 and SA exhibited bacterial growth inhibition of up to 10 and 8 h, respectively, at MOI 1 (Chang et al., 2015; Hamza et al., 2016). Other *S. aureus* phages UPMK_1 and UPMK_2 inhibited bacterial growth for 2–3 h at MOI 1 (Dakheel et al., 2019). PALS2 also showed a relatively fast bacterial inhibitory effect compared to the other jumbo phages that have been characterized. For example, bacterial inhibition was only observed until 200 min after infection by *Pseudomonas aeruginosa* phage MIJ3 (Imam et al., 2019) and 11 h after infection by *Cronobacter sakazakii* phage CR5 (Lee et al., 2016). Therefore, these results suggest the possibility of phage PALS2 as an effective *Staphylococcus* countermeasure (Figure 2).

### Host Range of Phage PALS2

To determine the antimicrobial spectrum of phage PALS2, 14 strains of *S. aureus*, 11 strains of staphylococcal strains other than *S. aureus*, and four additional Gram-positive strains were analyzed, as shown in Table 1. Notably, PALS2 was able to infect all *S. aureus* strains tested, including MRSA strains. Phage PALS2 was also capable of infecting other staphylococcal strains, including *S. haemolyticus*, *S. hominis*, *S. warneri*, *S. saprophyticus*, *S. capitis*, and *S. cohnii*. The wide antimicrobial spectrum of phage PALS2 suggests its potential applicability as a biocontrol agent in clinical settings, but further research is required to determine a more comprehensive host range of PALS2 against *S. aureus* strains by identifying phage resistance mechanisms in a hierarchical manner.

### Receptor Analysis of Phage PALS2

**FIGURE 3:**
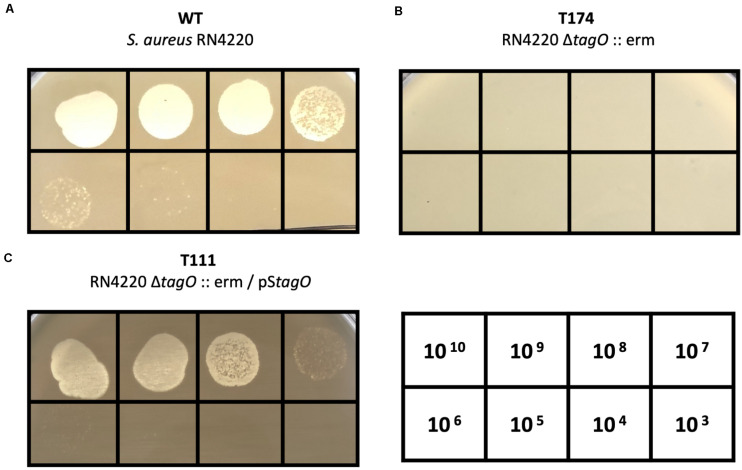
WTA-dependent infection of *S. aureus* phage PALS2. Phage PALS2 lysate was spotted onto lawns of **(A)** wild-type RN4220, **(B)** Δ*tagO*:erm mutant, T174, and **(C)** *tagO*-complemented strain, T111. The numbers in the table indicate the titer (PFUs/mL) of phage PALS2 spotted on the plate. Plaque formation indicates successful adsorption and infection by phage PALS2.

It is known that peptidoglycan-anchored WTAs of *S. aureus* serve as receptors for *S. aureus*–targeting phages. A previous study suggested that most *Staphylococcus* myoviruses require WTA to infect host bacteria (Xia et al., 2011). Therefore, we tested the infectivity of PALS2 on the Δ*tagO* mutant of *S. aureus* (RN4220Δ*tagO*:erm) (Kaito and Sekimizu, 2007), which lacks WTA (Figure 3). RN4220Δ*tagO*:erm was determined to be resistant to PALS2 (Figure 3B), and phage susceptibility was recovered when the strain was complemented with the *tagO* gene (Figure 3C; Park et al., 2010). These results indicated that WTA is the host receptor of phage PALS2.

### One-Step Growth Curve of Phage PALS2

**FIGURE 4:**
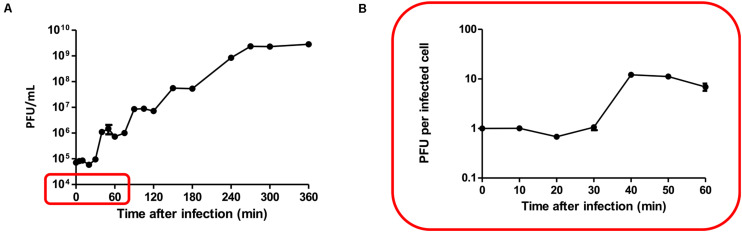
One-step growth curve. The data shown are the mean values from four independent measurements, and the error bars represent the standard deviations. **(A)** Multiple bursts of roughly the same size occurred approximately 30 min after each burst until the phage concentration reached its maximum, 10^9^–10^10^ PFUs/mL. **(B)** PALS2 demonstrated a latent period of 30 min followed by a small burst of progeny (12 PFUs per infected cell).

The latent period and burst size of PALS2 were determined by one-step growth curve analysis with the host strain *S. aureus* human isolate 93 (Hyman and Abedon, 2009). The latent period of PALS2 was 30 min followed by a small burst of 12 PFUs/infected cell (Figure 4). Multiple bursts of the same size occurred after an additional latent period of 30 min and were repeated until the phage concentration reached 10^9^–10^10^ PFUs/mL. Consistent with this result, multiple burst phenomena have also been observed in other phages (Shkoporov et al., 2018; Sharma et al., 2019). *Erwinia* jumbo phage Deimos–Minion showed double bursts of approximately five PFUs/infected cell, and *Bacteroidales* phage ΦcrAss001 showed two small bursts of 2.5 PFUs/infected cell. These phages commonly have small burst sizes, which could provide an explanation for the multiple bursts exhibited by jumbo phages, including PALS2. A previously reported study demonstrated that the small amounts of phages that come out of initially infected cells proceed to infect other intact cells in the culture, resulting in a second lag period and a subsequent burst (Ellis and Delbruck, 1939).

The small single burst size of PALS2 indicates low phage productivity, but the phage replication capacity is expected to be redeemed through repeated bursts. The high replication capacity of PALS2 could also be verified in the bacterial growth inhibition assay, where inhibition of *S. aureus* persisted for up to 24 h after infection with MOI 0.1, which is the same MOI condition given in the one-step growth curve assay.

### Whole Genome Analysis of Phage PALS2

Genomic features of phage PALS2 were elucidated by whole-genome analysis. PALS2 is a double-stranded DNA virus with a 268,748-bp-long chromosome and is thus classified as a jumbo phage (Yuan and Gao, 2017). The average G + C content of the genome is 24.66%, and it contains 279 ORFs that could encode proteins and one gene encoding tRNA with asparagine anticodon. The majority of the predicted genes (232 ORFs) encoded hypothetical proteins with unknown functions (Supplementary Table 1). No genes of predicted lysogeny functions, such as integrase, transposase, excisionase, repressor, and genome attachment site (attP), were identified in the genome of PALS2, suggesting that PALS2 is a lytic phage (Altamirano and Barr, 2019). Additionally, genes related to bacterial toxicity were not identified, but PALS2 encoded a metal-dependent hydrolase containing a metallo–beta-lactamase superfamily motif (PALS2_034). BlastN analysis demonstrated that the whole-nucleotide sequence of PALS2 shares less than 1% average nucleotide identity with other publicized phage genomes. Based on the low-genome homology between jumbo phages and other phages, many of them have been classified as a new lineage (Yuan and Gao, 2017). It is assumed that PALS2 is also a new phage species that does not belong to any other phage group.

**FIGURE 5:**
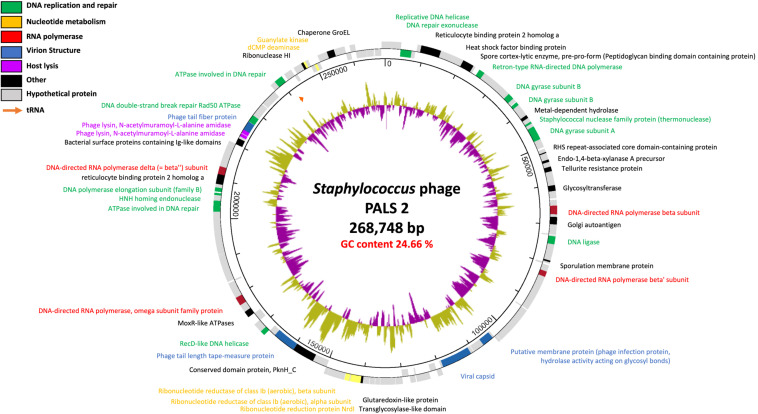
Genome map of phage PALS2. Predicted ORFs are arranged on the PALS2 genome. Functional groups are categorized into colors as shown in the legend. The inner track represents the GC content plot (500-base window) of the phage.

The large genome size of jumbo phages is believed to have evolved through the acquisition of additional functional genes (Yuan and Gao, 2017). PALS2 had 14 genes involved in DNA replication and repair and five genes responsible for nucleotide metabolism (Figure 5). Interestingly, four paralogous genes of the RNA polymerase (RNAP) subunit (PALS2_067, PALS2_089, PALS2_188, and PALS2_228) were identified in the PALS2 genome and were annotated as DNA-directed RNAP β, β′, ω, and δ subunits, respectively. When searched against the NCBI database, the annotated RNAP β, β′, and δ subunits showed significant biological similarities to those of various jumbo phages. The RNAP subunits of *Bacillus* phage AR9 exhibited the highest similarities with those of PALS2 (Lavysh et al., 2016): PALS2_067 had 91% coverage with 38% identity; PALS2_089 had 93% coverage with 37% identity; and PALS2_228 had 97% coverage with 32% identity. Meanwhile, the annotated RNAP ω subunit showed no detectable similarity to any protein in the NCBI database. Overall, it is assumed that the gene expression of PALS2 may be dependent more on its own RNAPs than on the host bacterial RNAPs (Ceyssens et al., 2014; Leskinen et al., 2016). This finding is also a probable explanation for the broad host range of PALS2.

PALS2 also encoded an additional gene involved in the lysis of host cells. This feature is expected to facilitate the infection ability of jumbo phages (Gill et al., 2012). Two genes (PALS2_239 and PALS2_240) were predicted as endolysins, which contribute to the hydrolysis of bacterial peptidoglycan. Endolysin is generally composed of two functional domains: enzymatically active domains (EADs) at the N-terminal position and cell wall–binding domains (CBDs) at the C-terminal position (Loessner, 2005). According to Pfam32.0 analysis, PALS2_239 was predicted to contain a cysteine, histidine-dependent amidohydrolases/peptidase domain (pfam05257; *E* value, 1.38e-07) for EAD and an SH3_5 domain (pfam08460; *E* value, 4.06e-08) for CBD. PALS2_240 was predicted to be composed of an amidase 2 domain (pfam01510; *E* value, 5.87e-20) for EAD and an SH3_5 domain (pfam08460; *E* value, 1.25e-08) for CBD.

Finally, PALS2 has an atypical genome arrangement, which is a common feature of jumbo phages. The genomes of small-genome phages such as a well-studied phage T4 are usually clustered into functional modules, and phage genes are expressed in a timely manner for the production of progeny phages. However, jumbo phages lack recognizable modular genome characteristics that help to classify this group of phages (Imam et al., 2019). Genes of associated functions in the genome of PALS2 were scattered or only formed subclusters, indicating that the majority of the genes are not expressed in a time-dependent manner but rather under control of the phage-encoded RNAPs (Leskinen et al., 2016). Overall, the genomic characteristics of PALS2 suggest that the large genome of this phage has evolved toward reduced reliance on the host bacterium. Accordingly, this genomic evidence, indicating a broad host spectrum and efficient lysis ability, suggests that PALS2 can be used as an effective therapeutic agent in the future.

### Comparative Genomics of Phage PALS2

**FIGURE 6:**
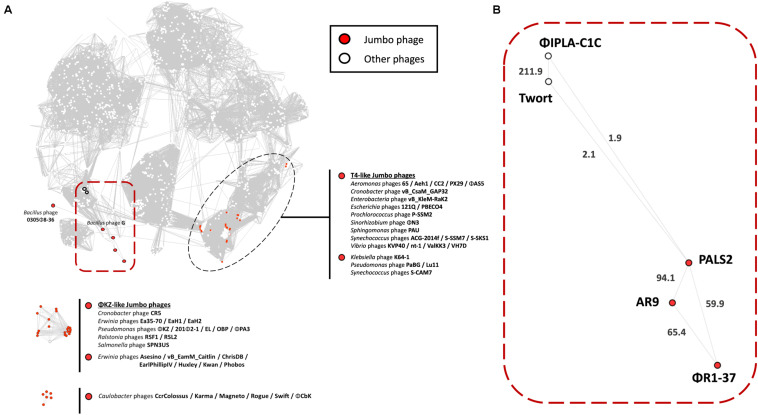
Protein-sharing network of phage PALS2. **(A)** A network representation was produced using Cytoscape. Nodes indicate phage genomes, and edges between two nodes indicate their statistically weighted pairwise similarities with phage–phage similarity scores of ≥1. Nodes are colored according to the phages’ characteristics: jumbo phage, red; other phages, white. Jumbo phages were further labeled with their designated names. On the top is the largest subnetwork that contains the largest number of phage genomes, including jumbo phages from the T4-like clade. The other subnetworks below are the smaller clusters of jumbo phages and their relatives that were disconnected from the largest subnetwork. Phage PALS2 and its first neighbors are situated in the large subnetwork, which is highlighted with a red square boundary. **(B)** Enlarged view of phage PALS2 and its relatives. Edge length is proportional to the similarity values estimated with the hypergeometric equation. The values are written on top of each edge. The data indicate that PALS2 is closer to jumbo phages AR9 and phiR1-37 than to the staphylococcal phages Twort and phiPLA-C1C.

To determine the location of PALS2 within the phage continuum context, we conducted a gene-sharing network analysis using vContact2 (Jang et al., 2019). This approach localized viruses sharing a high number of genes (homologous PC) into a VC. In the visualized network, the nodes indicate viral genomes, and the edges between nodes indicate the gene content similarities between each paired genome. Subnetworks that did not contain jumbo phages were removed from the gene sharing network to simplify the visualization, resulting in 1,446 nodes and 52,066 edges (Figure 6A). A full list of viral genomes used in the vContact2 analysis can be found in Supplementary Table 2. Of 53 jumbo phages identified on the map, 28 were placed in the largest subnetwork, including PALS2, and 25 others were placed into two isolated subnetworks. Conventionally, jumbo phages are classified primarily into T4-related or phiKZ-related phage groups. In the resulting map, the majority of jumbo phages in the largest subnetwork were related to the T4-like phages (Figure 6A). Notably, PALS2, which is placed in the large subnetwork, was distant from the T4-like phage group. The map indicated that PALS2 is connected to four *Myoviridae* phages: two *Staphylococcus* phages, Twort and phiIPLA-C1C, *Bacillus* jumbo phage AR9, and *Yersinia* phage jumbo phiR1-37 (Figure 6B). According to the similarity score estimated with the hypergeometric equation (Bolduc et al., 2017), PALS2 was more strongly connected to the jumbo phages than to the *Staphylococcus* viruses. This finding indicates that PALS2 shares more genes with the jumbo phages than the two staphylococcal phages. The result of the comparative genomic analysis using vContact2 explains the acquisition of many new genes as PALS2 evolved into a jumbo virus and thus suggests that PALS2 is more similar to typical jumbo phages.

In a previous study, phages AR9 and phiR1-37 were reported to be phiKZ-related, given that they possess genes encoding proteins homologous to the subunits of cellular RNAPs (Lavysh et al., 2016). The RNAPs of phiKZ-related phages are distinct from other phage RNAPs in that they are evolutionarily related to cellular RNAPs and belong to the double-psi beta-barrel fold family of polymerases (Yakunina et al., 2015). RNAPs of phiKZ-related phages, however, are not equal to their cellular counterparts, as they do not harbor the RNAP α subunit, nor do the phages encode bacterial sigma factors. BlastP analysis demonstrated that PALS2 encodes a unique multisubunit RNAP, and each subunit, except for PALS2_188, is significantly related to phages AR9 and phiR1-37, suggesting that it is possible that PALS2 is also a phiKZ-like virus.

## Conclusion

This research elucidated the physiological and genomic characteristics of a novel *Staphylococcus* jumbo phage PALS2. PALS2 exhibited a wide host range spectrum covering many species of *Staphylococcus* including MRSA. Therewith, the strong bacterial inhibitory activity of PALS2 demonstrated that the phage itself can potentially serve as an effective biocontrol agent. Genome exploration demonstrated that PALS2 possesses many extra functional genes, such as RNAP subunits and an extra lysis protein, which may have strengthened the infection ability of PALS2 and broadened its host spectrum. These observations place PALS2 among candidate phages to study their therapeutic potential in fighting infections with staphylococci. Further research regarding phage–host interactions would allow better insight into this jumbo phage.

## Data Availability Statement

The raw data supporting the conclusion of this article will be made available by the authors, without undue reservation.

## Author Contributions

YL, BS, YC, and SR conceived and designed the experiments. YL carried out the main body of research, performed the experiments and bioinformatics analysis, and wrote the manuscript. BS contributed in performing the phage characterization experiment and wrote the manuscript. YC contributed in isolating and characterizing the phage. SR supervised the work progress and edited the manuscript. All authors read and approved the final manuscript.

## Conflict of Interest

The authors declare that the research was conducted in the absence of any commercial or financial relationships that could be construed as a potential conflict of interest.
