# Supplementary material for: Characterization and Genomic Analysis of PALS2, a Novel Staphylococcus Jumbo Bacteriophage
Source: Front Microbiol. 2021 Mar 8;12:622755. doi: 10.3389/fmicb.2021.622755 (PMC7982418; doi:10.3389/fmicb.2021.622755)
Supplement: Supplementary file 1 [file Presentation_1.pdf]

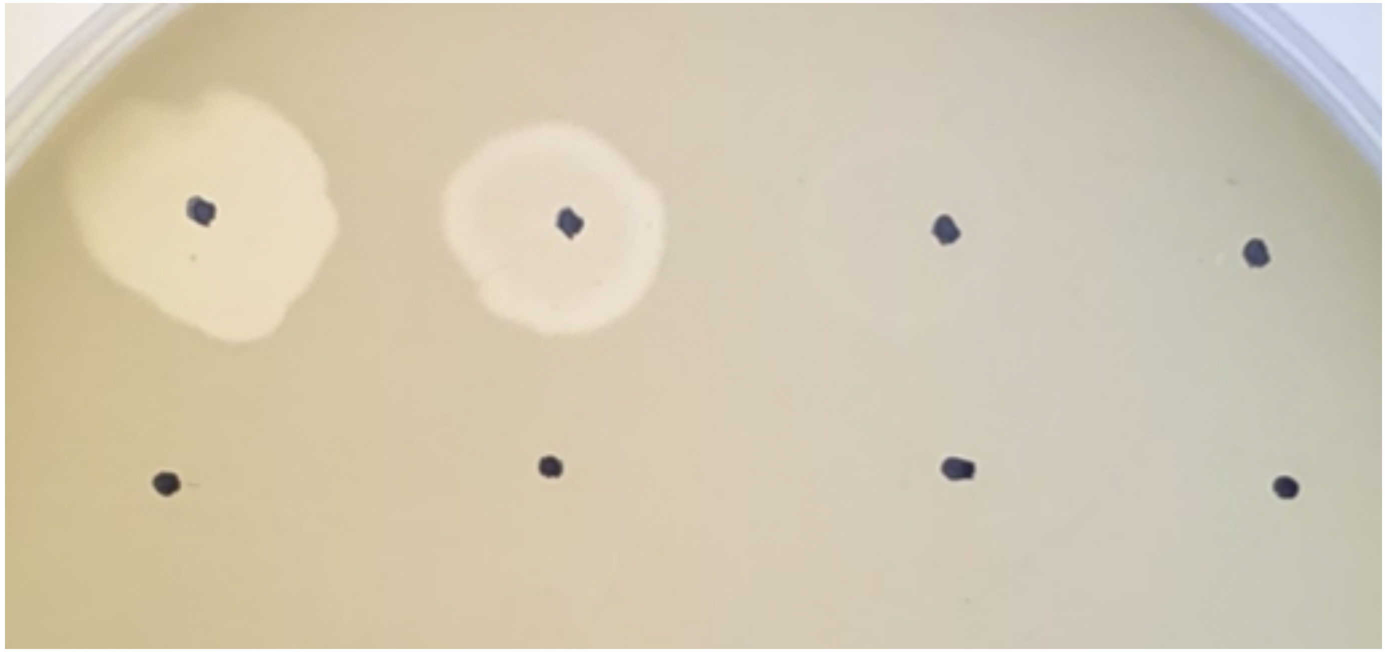

Supplementary Figure 1. Inhibition zone of phage PALS2. A photograph of phage inhibition zone. Inhibition means that the host growth on soft agar is inhibited due to 'lysis from without'. Since the phage did not propagate in the host, no single plaque is observed.
